# Market Access Advancements and Challenges in “Drug-Companion Diagnostic Test” Co-Development in Europe

**DOI:** 10.3390/jpm5020213

**Published:** 2015-06-12

**Authors:** Ildar Akhmetov, Rakshambikai Ramaswamy, Illias Akhmetov, Phani Kishore Thimmaraju

**Affiliations:** 1 Phamax Analytics Resources Pvt. Ltd. #19, KMJ Ascend 1st Cross, 17th C Main 5th Block, Koramangala Bangalore 560 095, India; E-Mails: ildar.akhmetov@phamax.ch (I.A.); raksha.svr@gmail.com (R.R.); 2 Unicorn, POB 91, Zhytomyr 10020, Ukraine; E-Mail: ia@unicorn-exp.com

**Keywords:** Stratified medicine, companion diagnostics, market access, cost-effectiveness, value addition

## Abstract

The pharma ecosphere is witnessing a measured transformation from the one-size-fits-all or blockbuster model of drugs to more informed and tailored personalized treatments that facilitate higher safety and efficacy for a relevant sub-population. However, with several breakthroughs still in a nascent stage, market access becomes a crucial factor for commercial success, especially when it comes to co-creating value for pertinent stakeholders. This article highlights diverse issues from stakeholder perspectives in Europe, specifically the ones which require immediate resolution. Furthermore, the article also discusses case studies articulating potential solutions for the issues discussed.

## 1. Introduction

High variability in alleles of human genes coupled with the heterogeneity of disease patterns signify the uniqueness of each and every pathological case requiring a more targeted approach to treatment.

At present, it is evident that 90% of all conventional remedies are not efficacious in 50%–70% of cases, and in the areas of oncology and neuroscience, approximately 25%–62% of patients fail to respond to standard therapeutics [1,2]. Moreover, the traditional “one-size-fits-all” clinical decisions frequently cause serious adverse drug reactions (ADR), which amount for 7%–13% of all hospital admissions in a number of developed European countries, like the UK and Sweden [3].

In the quest for new clinical solutions, tailored towards patients’ specific circumstances, pharmaceutical companies have announced the combination of therapeutics (Rx) and companion diagnostics (CDx), defined to increase the amount of potentially safer and efficacious treatments, facilitate early diagnoses of diseases and improve quality of life of millions.

Although the combination of drugs and companion diagnostics bears greater potential value for patients, physicians and health economics, it also increases the complexity of development, commercialization and regulation of therapeutics, resulting in limited access of these innovative products to patients. Thus, in 2013, the European CDx market accounted for less than 5% of the total *in-vitro* diagnostics (IVD) market (EUR 10.5 billion) and less than 0.04% of the total spending on healthcare in Europe [4].

Such a small market share is not only directly correlated to the novelty of the “therapy-test” concept, but also to an underdeveloped reimbursement policy, absence of value-based public sector pricing, clinical trial challenges, cumbersome and dissonant approval guidelines, low public awareness on “Rx-CDx” combinations *etc*. Nevertheless, the days are not far off when companion diagnostics in combination with highly effective therapeutics will rupture the market in quest for “niche” blockbuster revenues. Today, close to 50% of the early stage pipeline assets of pharmaceutical companies involve the use of specific biomarkers, primarily in the field of oncology, immunology and neurodegenerative diseases. In Germany alone, over 35 therapeutic solutions associated with CDx, received marketing authorization as of December 2013 [5].

To realize the potential of “therapy-test” pairings, companies have to revise their market access strategies, creating value for all stakeholders at various institutional levels (patients, physicians, payers, policy makers *etc*.). This requires thorough understanding of stakeholders’ needs and careful study of critical points in the “therapy-test” product lifecycle with an ultimate goal—to accelerate patients’ access to quality “Rx-CDx” pairings without any reimbursement delays and additional costs.

This study aims at understanding the major advancements and challenges associated with “Drug-Diagnostic” pairings, which facilitate or impede the access to stratified medicine.

## 2. Definition of Market Access

**Figure 1:**
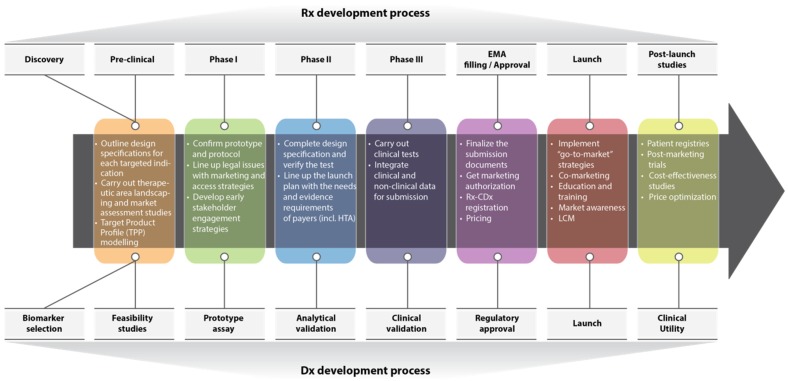
Perfect “Rx-CDx” co-development scenario in the context of market access. Adapted from Kostas *et al* [6].

In “therapy-test” combination, market access assists the right patients to get timely and easy access to a combined drug-diagnostic product at an affordable price. This involves engagement of multiple stakeholders facilitating key events in policy shaping during a product lifecycle to enhance drug accessibility (Figure 1).

Companies usually start drafting the preliminary market access strategies already at the early stage of a “therapy-test” co-development process to guide investment decisions and to ensure that variability in time and nature of diagnostics and pharmaceutical development platforms do not cause any delays or failures in bringing the combined product to the market. From the regulatory perspective, such drafts must provide insights on the legal requirements for marketing authorization, timely preparation of “Rx-CDx” product dossiers as well as “global” conformity in standards and labeling. Apart from that, principal concerns of market access during the “therapy-test” co-development and commercialization processes are creation, substantiation and communication of value to stakeholders via health technology assessment (HTA), health economics and outcomes research (HEOR), pricing and reimbursement, clinical trials and patient registries both in the pre and post launch periods.

The bottom-line objective of the market access function is to achieve commercial success by means of co-creating value for all relevant stakeholders.

## 3. “Rx-CDx” Co-Development

The co-development of a therapeutic agent and a companion diagnostic test is an important part of the combined “Rx-CDx” product lifecycle, which has a tremendous impact on the robustness of value proposition, hence paving the way to overall market access success. A well-aligned co-development program has a potential to leverage patient selection strategies to reduce clinical study size (optimize costs and accelerate the trial completion), enhance the safety profile, improve efficacy and increase the chance to achieve earlier regulatory submission and launches.

The study of the risk of clinical trial failure in non-small cell lung cancer (NSCLC), based on data from 676 trials, showed that only 11% of regular medicines had passed all three phases of clinical testing and received marketing authorization, while biomarker targeted therapies were found to have the highest success rate of 69% [7]. Apart from this study, many scholars also suggest that the use of biomarkers reduces the overall attrition rate and simplifies the regulatory approval process [8,9]. For example, Roche, in partnership with Plexxikon, developed the biomarker-based Cobas™ 4800 BRAF V600 Mutation Test, which enabled the marketing authorization of Zelboraf in less than six years from the discovery time of vemurafenib, while in average it takes 10–15 years for a drug to reach the market [10].

Since diagnostics are required at different stages of pre-clinical and clinical studies, it is important for the Dx and Rx companies to synchronize their development programs to ensure complete understanding of common objectives, timeliness and cost-risk optimization patterns. Partners should align and continuously develop target product profiles (TPPs) of an agent and diagnostics to articulate the technical, clinical and commercial performance of the test that will ensure successful development of a linked stratified medicine [8]. A well-developed TPP should be the guide for strategic clinical development decision-making and involve enough criteria of the agent’s actual clinical profile vis-à-vis the integral characteristics of a potentially successful product.

The early biomarker discovery projects facilitate the fast launch of the co-development strategy and allow companies to test hypotheses around pharmacodynamics and potential options for patient selection in phase I–II. If the biomarker hypothesis is promising, the assay passes through the analytical validation and estimation of the threshold value, based on data derived from outcomes from the early clinical trial phases. Implementation of the validated biomarker-based assay lets companies develop early patient enrichment strategies to enhance the success rate of Proof of Concept in a stratified patient cohort.

According to the European Commission, qualification and validation processes, as well as clinical and laboratory procedures, are critical for the co-development of proper CDx tests (tissue collection, standardization of technologies, prospective clinical trials *etc*.) [11]. The Commission suggests that the following requirements are considered as a standard for successful approval of a CDx:

- High analytical validity;
- Appropriate sensitivity and specificity;
- Clinical validity/ Clinical utility;
- Ability to influence treatment plan;
- Ethical and social acceptance [11].

Despite the time and organizational challenges in developing the Rx and CDx assay, experts do not recommend beginning the clinical trial with a prototype assay and replacing it with the validated version later during the trial, as it creates difficulties in interpreting study results, because the patients get selected based on two different versions of the assay [12]. It is also important for the partners to always keep in mind the purpose of the latest clinical trial phase, *i.e*., not just to demonstrate safety and efficacy of the therapeutic agent, but to clinically validate the predictive potential of the CDx test with respect to an individual patient [12]. Finally, companies have to retain that the regulatory acceptability of excluding biomarker-negative patients from trials depends on the strength of evidence (plausibility, scientific rationale and clinical data) provided based on the lack of effect in these patients [13].

It is important for CDx and Rx partners to receive timely consultations from the European Medicines Association (EMA) or a national competent authority on the suitability of the test versus safe and effective use of the therapeutic product in question. Such consultations will contribute to better understanding of the depth of evidence required for marketing authorization, hence facilitate market access of the “therapy-test” combination.

In the history of personalized medicine, Roche/Genentech was the first company to successfully use the “therapy-test” co-development model, bringing Herceptin® (trastuzumab, Genentech-Roche, San Francisco, USA) to the market in 1998 along with Dako’s immunohistochemistry assay HercepTest™ (Dako, Agilent Technologies, Santa Clara, USA), targeting HER2 overexpression in advanced breast cancer. This prompted numerous novel targeted cancer medicines guided by a CDx assay to seek the approval of the US Food and Drug Administration (FDA) and European Medicines Agency (EMA), including cetuximab, crizotinib, vemurafenib *etc.* [14]. Companies recognize enormous value in “therapy-test” co-development and its impact on the commercial triumph of new therapeutic solutions. Albeit, the well-thought-through co-development process is not the only critical point in market access success, since there are additional vital considerations like regulatory and labeling, HTA and reimbursement, pricing and post-launch requirements *etc*.

## 4. Regulatory and Labeling

Presently, there is no coordinated mechanism to assess drug-diagnostic companion products in Europe, because of tremendous differences in regulatory guidelines for medicines and tests, which lead to inconsistent decision making at the EU level, hence hindering market access.

From a drug manufacturer perspective, there are two ways of getting a marketing authorization for medicines in Europe—decentralized and centralized. The decentralized procedure is limited to a single member state and requires manufacturers to submit marketing authorization applications (MAA) to the national competent authorities. The centralized procedure for the initial assessment of new drugs seeking the EU-wide marketing authorization is conducted by the Committee for Medicinal Products for Human Use (CHMP), an integral part of the European Medicinal Agency (EMA). The CHMP prepares the EMA's opinions on all questions concerning medicines for human use, based on purely scientific evaluation of quality, safety and efficacy profiles, in accordance with Regulation (EC) No 726/2004 and Directive 2001/83/EC. Post EMA’s assessment of the MAA, it is the European Commission that approves or rejects the marketing authorization of a medicine in the EU.

In both procedures, the regulatory authorities require that an IVD companion diagnostic device is stipulated in the labeling of a therapeutic. However, this label does not specify which particular diagnostic device should be used as a CDx. Hence, it grants permission to any validated test, which complies with Directive 98/79/EC with regards to safety and clinical utility, to be applicable with an assigned therapeutic. Considering that all *in-vitro* tests used for the same biomarker vary in methodology, validation criteria and targeted mutations, they are likely to have diverse effects on the stratified patient populations, hence causing significant deviations in health outcomes and benefit/risk ratio of the patients [5].

*In-vitro* diagnostic tests do not have pre-market approval, but a direct CE (Conformité Européene) mark authorization, which is granted based on self-certification of CDx manufacturers [15]. This procedure is regulated by the IVD Directive 98/79/CE, which contributes to the delivery of diagnostic tests across the European markets in a harmonized fashion [16]. The European Commission has a list of appointed independent third parties (notified bodies), which are the private organizations accredited to assess the IVD medical devices and grant CE mark certificates or declarations of conformity.

Main principles:

1. CE Marking on a product is a manufacturer’s declaration that the product complies with the essential requirements of the relevant European health, safety and environmental protection legislations, in practice by many of the so-called Product Directives.
2. CE Marking on a product indicates to governmental officials that the product may be legally placed on the marketin their country.
3. CE Marking on a product ensures the free movement of the product within the EFTA & European Union (EU) single market (total 30 countries), and
4. CE Marking on a product permits the withdrawal of the non-conforming products by customs and enforcement/vigilance authorities.

The aforementioned discussion suggests that the lack of uniformity in “therapy-test” approval may impede the achievement of the ultimate goal of personalized medicine—to deliver the right treatment to the right patient in the right time—and create substantial market access barriers for the original test used in the clinical trials.

### Future Considerations

Based on evaluated divergences in the interpretation and application of the IVD Directive 98/79/CE, the European Commission initiated a fundamental revision of the regulatory framework for *in-vitro* diagnostics, necessary to ensure a high level of human health and safety, facilitate competitiveness of the IVD industry, promote innovation and allow rapid cost-effective market access for innovative Dx [15].

The revised IVD legislation defines a companion diagnostic as “a device specifically intended to select patients with a previously diagnosed condition or predisposition as eligible for a targeted therapy” [17]. The new EU IVD guidelines are also set to empower notified bodies to apply the “New Approach” to conformity assessment, based on the novel classification of risk (A, B, C or D). The robustness of such IVD assessment and the requirement to demonstrate scientific validity, analytical and clinical performance are correlated with the level of risk of an individual Dx test – the higher the risk, the stricter is the manufacturer oversight.

Following the European Commission’s initiative, it is anticipated that close to 90% of all IVDs will be subject to some level of control by notified bodies [17]. Also, considering that CDx generally belongs to “Class C” risk category, the full involvement of the authorized bodies in the assessment process of these Dx tests is required. Additionally, consultations with national competent authorities or EMA is also mandatory.

Regulatory authorities should adequately recognize CDx as a powerful tool to achieve the objectives of personalized medicine, and provide consistent legislative support to Rx and Dx companies to facilitate patients’ accelerated access to innovative targeted therapies.

## 5. HTA, Pricing and Reimbursement

Growth in healthcare spending has tightened the budgets of all European member states, raising concerns regarding value for money in healthcare and leading to new avenues of cost-effectiveness and redefinition of “value”.

**Figure 2:**
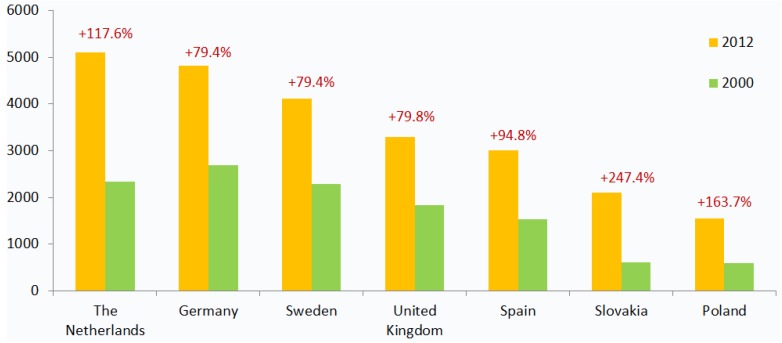
Growth in health expenditure per capita in select European markets, 2000–2012 (USD PPP). This figure shows an increase in healthcare expenditure per capita (in USD, purchasing power parity) between 2000 and 2012. The growth in healthcare spending signifies the financial pressure on state budgets and a need for novel cost-effective therapeutic solutions [18].

In countries like Germany, the UK and Sweden, the per-capita expenditure on health has increased by almost 80% since 2000, while in the Netherlands, Poland and Slovakia, this index has grown more than two times (Figure 2) [18]. As a proportion of GDP, healthcare spending has exceeded economic growth in almost all European countries in the past fifteen years.

In light of increasing budgetary constraints and high out-of-pocket expenses of patients, health technology assessment (HTA) becomes one of the prominent tools obligatory for a transparent, non-biased basis for decisions on the uptake of a medicinal product [11]. HTA platforms evaluate the costs, effectiveness and broader impact of healthcare solutions for those who plan, provide or receive care, taking into account clinical, social, economic and ethical issues [19].

### 5.1. Payers’ Perspective

From the public health systems’ and private insurers’ perspective, HTA is a mechanism to check whether an evaluated product adds value and is as effective in terms of costs and clinical benefit as the existing medicines from reimbursement lists. But, in case of a “therapy-test” pairing, there are literally no benchmarks, because in principle there are very few combination products that have received marketing authorization in Europe. Therefore, in spite of the fact that many tests displayed excellent utility in stratification of populations, there is a gap in clear evidence of their health outcomes impact.

Payers are apprehensive about the costs of testing a large “at risk” population to identify patients who may benefit from a particular medicine or require a change in dosing regimen, simply because they do not see a potential return on investment [20]. Also, even though diagnostic tests prove to be affordable (between EUR 30 and EUR 400 per test in average), paying for the entire population in many cases is less economically viable.

Nevertheless, the clinical practice contains case studies which prove the opposite. In a famous example, which involved stratifying sub-population of trastuzumab-receiving patients, both NICE (The National Institute for Health and Care Excellence) and SMC (Scottish Medicines Consortium) did not consider Herceptin to be cost-effective for the large gastric cancer population initially, but once the HER2 overexpression (IHC 3+) subgroup was defined, the drug was immediately considered as cost-effective. Additionally, the use of HLA-B*5701 testing in HIV infected patients initiating abacavir demonstrated a cost-effectiveness ratio of USD 36,700 per QALY (Quality Adjusted Life Year) compared to no testing [21]. Moreover, treatment using cetuximab only for patients with tumors who expressed wild-type KRAS showed more favorable cost-effectiveness ratio than treating patients irrespective of tumor KRAS mutation status [22]. Various parameters were considered in tabulating costs including direct drug costs, outpatient visits, surgical procedures and hospitalizations, adverse effects, tests and screening for KRAS mutations in a Canadian context.

Still, the cost-effectiveness may not apply to all diagnostics, especially if the prevalence of the marker is not very high. A study on treatment for NSCLC using crizotinib [23], revealed a dominant effect of screening cost per person by different methods at low biomarker frequencies. The specific model was later used to develop a general equation to predict cost-effectiveness on the basis of biomarker frequency. There was an exponential fall based on an increase in the frequency of the biomarker. For example, cost-effectiveness of <USD 100,000 per QALY gained is not achievable at biomarker frequencies <5% (with drug costs USD 1–5000 per month and screening costs USD 600–1,400 per person).

A key reflection, which helps companies and payers to better understand the rationale in developing and reimbursing personalized medicines is minimization of over-treatment and drug wastage costs. Drugs which are unsafe or ineffective cause avoidable deaths and adverse reactions, many of which lead to hospitalizations costing logistic wastage of resources; and drug wastage that results from discarding medications that do not function [24,25]. As per the National Health Service (NHS), the estimated cost of unused/partially used prescription drugs is GBP 300 million annually [26], which indicates a huge financial burden on the budgets of payers, augmenting their keen interest in personalized medicine.

Likewise, some treatments lead to adverse reactions (an estimated societal cost of EUR 79 billion/year, according to the EU commission) [27]. For example, the variant CYP2C9 gene alters the rate of warfarin metabolism (a commonly used anti-coagulant drug in treatment of coronary heart diseases). The variant has a higher rate of occurrence in Caucasian subgroups in comparison to Afro-Caribbeans or Asians. The result of this variant is slower metabolism of the drug, requiring lesser dosage. High dosage was reported in severe complications such as hemorrhage, experienced by 8% to 26% of patients treated for at least one year [28]. Development of pharmacogenetic testing for CYP2C9 alleles is underway as part of a GBP four million project by the UK government to identify people at risk of warfarin associated bleeding and certain drug interactions [29]. 

To address some of the aforementioned challenges arising because of personalized medicine, companies nowadays turn to public and private collaborative practices, which in many ways appear to be successful. One such collaborative program involved the Dutch healthcare insurer CZ, the Center for Personalized Cancer Treatment (CPCT) and VitrOmics, a company specialized in personalized medicine. The three combined received a “Value Based Healthcare Encouragement Award” for their joint project to address the overtreatment issue within a defined group of breast cancer patients by providing therapy in a much more selective way than in clinical practices before [30].

Such practices enable stakeholders to estimate the thresholds of benefit at which therapies are effective enough to justify funding. But, these thresholds must also consider the impact of a therapy on the broader community (patients, physicians, payers, health economics *etc.*) along with potential consequences of paying for a single person treated with a high cost therapy versus providing less expensive services to a larger number of people.

### 5.2. Differences in HTA and Reimbursement Systems

In the heterogeneous European environment, there are radical differences in HTA and reimbursement systems, which predefine diversity in budgetary allocation and reimbursement practices at the member state level. Some countries, like the UK, base their reimbursement decisions on cost-utility analysis by estimating the cost of interventions vis-à-vis the obtained health benefit (e.g., QALY) [16]. Other countries, like France and Germany, account for clinical added value, followed by “value for money” pricing debates.

**Figure 3:**
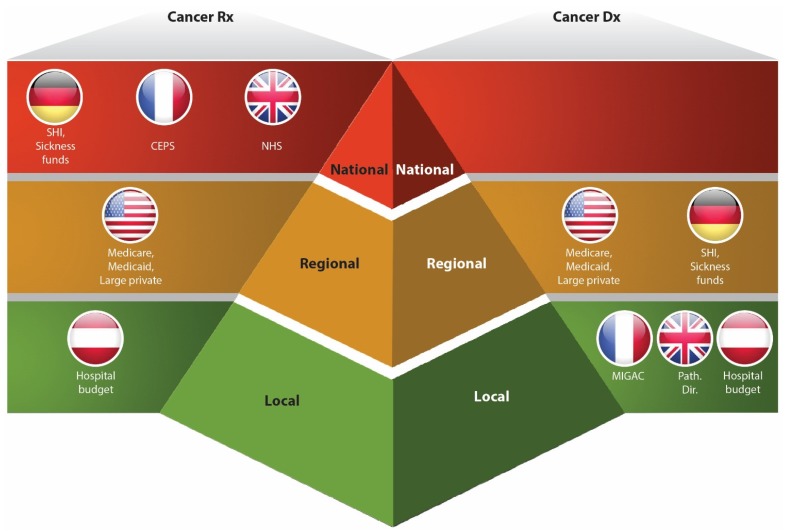
This highlights key differences in reimbursement policy approaches for oncology diagnostics and therapeutics. In most of European countries, the coverage for Dx tests occurs at the local and regional levels, while Rx is primarily reimbursed at the national level (except Austria). Such discrepancy creates additional challenges for “therapy-test” producers, as they have to approach miscellaneous payers using different clinical benefit and cost-effectiveness data sets. Adapted from [2,5].

The diversity in HTA systems of the EU member states is further complicated by centralized versus decentralized assessment and reimbursement processes. In most European states, coverage for prescription medicines is done at the national level, while diagnostic manufacturers have to approach local payers to win reimbursement for their companion tests (Figure 3). For example, Herceptin (tratuzumab) is commonly reimbursed in most of European member states, while its companion diagnostic test HER-2/neu is covered differently in each particular country. Thus, in the UK, Germany and Italy, the test is publicly funded, while in Spain it is a therapeutic partner who covers the expenses related to testing. In France HER-2 test was authorized in 2000, but has only been reimbursed since 2007 [31].

Moreover, depending on the product lifecycle stage, the approaches to HTA also vary. For example, at the pre-marketing authorization phase, HTA is focused on the first pricing and reimbursement decisions. Hence, it involves data related to the Phase I–III clinical trials and the related economic evaluations. In the post-launch period, such assessments involve more of “real world” data to support price optimization and reinforce/expand reimbursement decisions.

In the recent survey among companies dealing with HTA, the interviewed SME businesses acknowledged that the main obstacle in the reimbursement process for their therapeutics was the absence of adequate understanding of HTA specific requirements and principles [16]. It has been also observed that a great share of present day HTA procedures get negatively affected by the dearth of methodological agility and superfluous bureaucracy, thus causing delays in the identification of important genetics variants, poor description of trial designs, insufficient representation of patient experiences *etc*. [16].

As a result, in order to improve timely access of patients to new medicines, several revolutionary approaches to market access have been introduced:

• Adaptive pathways (former “adaptive licensing”) are a novel way to reduce uncertainties and to facilitate initial approval of a drug in a well-defined patient subgroup with a high unmet medical need under cautiously controlled conditions.

• Managed entry agreements (“risk sharing agreements”), introduced to simplify the market access process, represent formal arrangements between payers and manufacturers, aimed at sharing financial risks due to uncertainty related to the introduction of new technologies (e.g., price-volume agreements, outcome guarantee, coverage with evidence development and disease management programs) [32].

The emergence of such revolutionary new solutions to market access is an important milestone in developing new generation payer-producer relationships based on open dialogue, collaboration and co-creation of value for multiple stakeholders.

### 5.3. Value and Pricing

The vast majority of today’s pricing and reimbursement decisions are made during the launch phase, based on the data derived from randomized control trials, which measure the clinical effect of a therapeutic on an average patient. Since a “therapy-test” pairing assumes a more targeted approach to dosing and treatment, it allows better stratification of patients, confines medicines that do not work and leads to better compliance. Preliminary testing with CDx allows payers to cover the treatment only for those individuals who respond positively to a therapy. However, in light of the overall uncertainty regarding the definition of “value” for an innovative drug, payers remain conservative in their reimbursement decisions.

The lack of clear definition of “value” also prompts reimbursement and funding decision makers to prioritize cost-based pricing over value-based pricing. But, such approach obviously does not reward innovation, and considering the high costs of developing a companion diagnostic, Dx companies feel uncertain over eventual agreement on the reimbursement status.

Historically, pricing decisions for Rx and Dx are carried out separately. While pricing of a therapeutic is product specific, and it may imply reference pricing throughout the borders, companion diagnostic pricing is usually aligned with hospitals and laboratories, or is related to code-specific fee schedules [31]. Significant differences in pricing decisions are also observed in private and public healthcare sectors. While in the European private sector, value-based pricing for therapeutics has been practiced for long, the public sector is widely known for poorly developed pricing on the basis of value, and even the procedurally based pricing that presently exists is administered by a complex network of largely decentralized bodies [2]. Moreover, there are no value-based pricing guidelines for innovative diagnostics in any of the EU member states, and the outcome of such underdeveloped pricing system is the limited access of patients to novel “therapy-test” products and unrealized health-economic savings.

In the last couple of years, countries facing budgetary constraints, like Portugal, Greece and Spain, have undertaken mandatory price cuts for expensive therapeutics. A number of other countries, like Germany, have restructured their pricing and reimbursement systems. This suggests that pricing and reimbursement mechanisms in Europe are still in the process of formation and companies have to closely collaborate with decision makers in order to lobby their interests and co-develop pathways that would be mutually beneficial.

Several studies of the economic uplift of “therapy-test” combinations showed that despite a wide disparity in relative markets and pricing, the economic impact of the CDx to a “therapy-test” co-development program, in terms of the contribution to the net present value (NPV) of the combined Rx-CDx product, is projected to increase from USD 900mn to USD 2.7bn over a twenty year period, which is substantial [8]. But, as long as this value is not reflected in the clear evidence for payers with detailed specifications of economic and monetary benefits, there is no way to bridge “Rx-CDx” partners and reimbursement authorities. Such value must integrate clinical benefits and real-world evidence demonstration with timely incorporation of medical innovation in patient care. A famous case study on the use of KRAS testing proves the possibility of winning reimbursement by generating powerful evidence that integration of genetic warfarin test could avoid 85,000 serious bleeding events and 17,000 strokes, offering an annual saving of USD 1.1 billion in the US [2].

## 6. Post-Launch Considerations

The post-launch strategies are important ingredients of the market access mix that may impede quick and smooth delivery of combination products to the market, if not addressed correctly. Early adoption of a product depends largely on a manufacturer’s ability to increase physicians’ and patients’ uptake and to generate timely post-marketing real-life data customized for each stakeholder’s requirement and expectation.

### 6.1. Physicians’ and Patients’ Uptake

Despite the favorable transformation processes and visibly changing perception of personalized medicine amongst the key stakeholders, the “Rx-CDx” uptake and adoption among European physicians and patients remain limited due to lack of evidence-based clinical experience of CDx and the novelty of the concept. It may take many years for “therapy-test” partners to educate physicians and incorporate companion diagnostic testing into clinical practice. The famous case of HER2 diagnostic testing in breast cancer proves that producers may possibly have to spend many years and substantial efforts to educate healthcare providers through general and specific public health campaigns, provision of information and technological support, improving physicians’ ability to involve patients, counselling of patient regarding outcomes *etc*.

In many cases, physicians are not committed enough to molecularly guided therapeutic decisions and sometimes they lack necessary expertise in molecular biology. Common misperceptions of physicians about benefits and price of companion diagnostics and personalized medicine also have a negative impact on the uptake of “therapy-test” combinations in Europe. For example, many healthcare providers believe that benefits of “therapy-test” pairings and personalized medicine are limited to oncology, without realizing the recent promising developments in immunology, cardiovascular, CNS and other therapeutic areas. There is also a general perception of high price associated with use of companion diagnostic, which has to be necessarily addressed by “Rx-CDx” manufacturers.

Companies should communicate their educative messages through open events in a multidisciplinary learning environment, where all physicians (not only medical specialists, but also interventional radiologists, pathologists *etc.*) can meet and share their experiences. It is recommended that discussion-based formats (round tables, workshops and small group seminars) take over traditional approaches such as presentations, face-to-face or online campaigns, given the higher retention rate (50% versus 20%) [16].

Collaboration with patient advocacy and support groups is another crucial component in helping to raise awareness of “therapy-test” combinations and stratified medicine. Nowadays, patient advocates are empowered to navigate many aspects of treatment, like selecting the right physician or finding appropriate clinical trials. Such “advocacy-producer” collaborations will facilitate patient engagement in the areas of improved health outcomes, contribute to informed decision making and result in better access to care.

### 6.2. Pharmacovigilance and real world evidence

The increasing demand for real world evidence by regulatory authorities, payers and healthcare providers forces “Rx-CDx” companies to apply new data considerations and revise designs of post-marketing studies. Often at the time of “therapy-test” launch, reimbursement authorities are not fully confident about the clinical and economic benefits of the “Rx-CDx” combination product. Hence, they enable the access to pharmacogenetic tests, provided that post-marketing real world evidence is generated and supports their reimbursement decisions.

The post-marketing support for a companion diagnostic is just as important as that of a therapeutic, since the complexity of CDx development process may be quite challenging in maintaining the same level of accuracy, sensitivity and specificity of a diagnostic test. For instance, Dako, the producer of EGFR immunohistochemistry test for patient’s eligibility for Erbitux® in colorectal cancer, reported 70% to 80% over-expression in the initial pivotal trials, while the post-marketing study showed less than 50% over-expression of EGFR [33]. As a result of such inaccuracy in reported over-expression rates, a large number of eligible patients did not get access to EGFR-targeted therapy.

Real world evidence studies, such as patient registries or post-marketing clinical trials [34], allow tracking of long-term safety outcomes, improve decision making in treatments, encourage product use, enable development of effective risk evaluation and mitigation strategies, generate evidence on health coverage and contribute to access to quality personalized therapy for a larger amount of patients.

## 7. Conclusions

Personalized therapies offer incredible and revolutionary opportunities in achieving improved health outcomes in patients. Although very promising, high costs feature as a key limitation for accessibility. Patient stratification is an important aspect where appropriate companion diagnostics can offer substantial value by guiding therapies towards those patients who are likely to obtain maximal health benefits and reduced side effects, and costs for those patients who are not likely to benefit from these therapies. Scenarios that provide maximum value of companion diagnostics need to be identified by comparing different alternatives. Stratified medicines establish risk-sharing in test development and trial operations to both large pharmaceuticals and companion diagnostic developers, thus introducing a regulatory strategy for diagnostic developers and building designated markets for final products. Currently, various parameters that affect market access are still nascent in Europe, warranting development of mutually beneficial pathways involving key stakeholders. But, with larger acceptance of highly-effective patient-stratified therapies, healthcare will move towards an era of predictive medicine than the currently existing reactive medicine, with the undoubted benefits of earlier treatment translating to better long-term outcomes.
